# Determination of Colistin B in Chicken Muscle and Egg Using Ultra-High-Performance Liquid Chromatography–Tandem Mass Spectrometry

**DOI:** 10.3390/ijerph18052651

**Published:** 2021-03-06

**Authors:** Harsh Kumar, Dinesh Kumar, Eugenie Nepovimova, Dasharath Oulkar, Anil Kumar, Ramiz Mohammad Rafi Azad, Subodh Kumar Budakoti, Navneet Kumar Upadhyay, Rachna Verma, Kamil Kuča

**Affiliations:** 1 School of Bioengineering & Food Technology, Shoolini University of Biotechnology and Management Sciences, Solan 173229, India; microharshs@gmail.com (H.K.); kumaranil@shooliniuniversity.com (A.K.); 2 Department of Chemistry, Faculty of Science, University of Hradec Kralove, 50003 Hradec Kralove, Czech Republic; eugenie.nepovimova@uhk.cz; 3 Food Safety Solution Center, FSSAI-Thermo Fisher Scientific, Indirapuram, Ghaziabad 201014, India; dasharath.oulkar@thermofisher.com (D.O.); ramiz.azad@thermofisher.com (R.M.R.A.); subodh.budakoti@thermofisher.com (S.K.B.); 4 School of Pharmaceutical Sciences, Shoolini University of Biotechnology and Management Sciences, Solan 173229, India; navneetqa@gmail.com; 5 School of Biological and Environmental Sciences, Shoolini University of Biotechnology and Management Sciences, Solan 173229, India; rachnaverma@shooliniuniversity.com

**Keywords:** chicken, colistin, ultra-high-performance liquid chromatography, mass spectrometry

## Abstract

Colistin, an imperative member of the polymyxin group, is a cationic peptide antibiotic. Itis also known as polymyxin E, but this peptide antibiotic has been forbidden for human consumption due to its high toxicity. Regrettably, this antibiotic is utilized as a feed additive and veterinary drug for animals. Due to the toxicity of colistin, the presence of its residue in the animal system represents a threat to human health regarding the consumption of meat, especially chicken. A novel method was proposed for quantifying colistin B in chicken muscles and eggs using ultra-high-performance liquid chromatography–tandem mass spectrometry (UHPLC–MS/MS). In this method, extraction of colistin B from samples was achieved by mixing the sample with acidified methanol:water (1/1, *v*/*v*), followed by centrifugation and filtration by a membrane filter excluding solid-phase extraction (SPE) clean up, as well as evaporation steps. The analysis was conducted by optimized liquid chromatography–tandem mass spectrometry (LC–MS/MS), and method performance was assessed in terms of the limit of quantitation, specificity, selectivity, precision, linearity and recovery in coherence with the guidelines of SANTE and the Commission Decision 2002/657/EC. The result obtained from the study showed the limit of quantitation (LOQ) as 10 µg Kg^−1^ for muscles and 5 µg Kg^−1^ for eggs, with acceptable recoveries along with precision. The linearity was plotted in the range of 5–25 µg L^−1^ (solvent) for egg and 10–50 µg Kg^−1^ (matrix-matched) for muscles. The result of average recoveries showed the value of 70–94% (3.3–12% relative standard deviation (RSD)) for chicken muscles and 88–107% (2.5–18.6% RSD) for egg samples, which meets the criteria for acceptability of method according to both SANTE and 2002/657/EC guidelines. This proposed protocol provides a cost-effective solution for food testing labs by reducing the cost of the sample preparation by 60% along with the time required for SPE cleanup. Further, the optimized method was also tested on real samples collected from nearby provinces in Solan city, Himachal Pradesh, India, and three out of 20 muscles were found to have colistin B in the range of 50–560 µg Kg^−1^.

## 1. Introduction

Colistin (polymyxin E) is a cationic peptide non-ribosomal antibiotic synthesized by *Bacillus polymyxa* subspecies *colistinus,* and is an active member of the polymyxin group [1,2]. Colistin is stated to be composed of approximately thirty different constituents. Out of all the components, colistin A (polymyxin E1) and colistin B (polymyxin E2) are the most predominant, accounting for ≥85% of colistin utilized in pharmaceutical products, which only differ because of the single carbon in the fatty acyl moiety [2]. Owing to high toxicity, this peptide antibiotic has been banned for human consumption, but still it is extensively used as a feed additive and veterinary drug in animal husbandry [3]. Worldwide, national health authorities have determined and established maximum residue limits (MRLs) for colistin in chicken muscles and eggs (Table 1). 

In 2015, a team of researchers from China identified the colistin-resistant bacterial strain and stated that the presence of the mcr-1 gene was responsible for the development of colistin resistance in bacteria as it gets transferred from one bacterial strain to another [4]. Considering the analysis and reports related to this, the Chinese government in 2016 banned the usage of colistin for livestock. This led to a huge loss to pharmaceutical industries producing colistin, as China alone was responsible for the demand of 8000 tons of colistin per year, while the global demand was 12,000 tons per year. In spite of the ban, Chinese agrichemical companies are the leading manufacturer and exporter of colistin in countries like Vietnam, India and South Korea [5]. 

In India, five pharmaceutical companies producing animal drugs promote products containing colistin for metaphylactic purposes, as well as for growth. According to an investigation conducted by the Bureau of Investigative Journalism of London, chickens of India showed the presence of heavy residues of antibiotics in their tissues. In India, Venky’s is the main seller of chicken products, and has reported the use of colistin in the livestock sector for therapeutic purposes [6]. These unregulated and non-monitored practices have led to the development of drug resistance in bacteria, and as per consensus, around 57% of bacteria (Gram-negative) in India alone are resistant to carbapenem. The Indian government has taken an initiative to prohibit colistin usage as a growth-enhancing supplement, but this initiative has failed to generate effective results due to a lack of association of government regulatory bodies in this initiative. Lately, the Food Safety and Standards Authority of India (FSSAI) has proposed to determine the antibiotics level (tolerance) in food products, and keeping this in focus in 2011, the standards governing contaminants, residues and toxins were revised [7]. In 2019, FSSAI put continuous effort into completely banning the use of colistin [8].

Even though colistin use is banned, there is still a need to monitor the colistin residue in animal origin foods. In preceding years, different liquid chromatography–tandem mass spectrometry (LC–MS/MS)-based methods were developed and used for investigating polypeptide antibiotics in animal origin food. For instance, Sin et al. [9] have reported the bacitracin A as well as the colistin A (50 µg Kg^−1^) detection methods in bovine samples. Wan et al. [10] reported the use of an acidified extraction procedure for pretreatment of animal tissue and milk samples prior to solid-phase extraction using Strata-Xcartridge for detection of colistin B in samples. Similarly, Xu et al. [11] and Boison et al. [12] published methods for detecting the presence of colistin A (40 µg Kg^−1^ in fish and 39.0 µg Kg^−1^ chicken muscle) and B in fishery products and chicken muscle, respectively. 

The aim of the present study was to develop a simple, cost effective and sensitive ultra-high-performance liquid chromatography–tandem mass spectrometry (UHPLC–MS/MS) method and validate it for colistin B residue determination in chicken muscle as well as egg samples in contrast to the earlier reported methods, which involve acidic extraction followed by solid-phase extraction clean-up procedures. Further, the validation of this newly optimized method was done as per the SANTE and the Commission Decision 2002/657/EC guidelines.

## 2. Materials and Methods

### 2.1. Chemicals, Reagents and Apparatus

LC–MS grade methanol and formic acid (98% purity) were procured from Fisher Chemicals (Hampton, NH, USA). Deionized water (18.2 MΩ) was obtained from the Bamstead^TM^MicroPure water purification system (ThermoScientific^TM^). Syringe filters, labeled, CHROMAFIL Xtra polyethersulfone(PES), 25 mm, 0.20 µm, polytetrafluoroethylene(PTFE), 25 mm, 0.45 µm and nylon, 25 mm, 0.45 µm were purchased from Macherey-Nagel (Düren, Germany). 

The reference standards of colistin sulfate (colistin was 753 µg mg^−1^, and colistin B was 53.6%, according to the certificate of analysis) were procured from Sigma-Aldrich, Bangalore, India. The stock solution (1000 µg mL^−1^) of analyte was prepared in methanol/1% formic acid in water (1/1, *v*/*v*) by correcting the sulfate mass, and kept at −20 °C. The working standards were prepared via serial dilution with methanol/1% formic acid in water (1/1, *v*/*v*). These working standard solutions were then used for preparing linearity and spiking studies. 

A refrigerated centrifuge Sorvall™ ST8 ventilated benchtop (ThermoScientific, San Jose, CA, USA), analytical balance (Aczet, CY2202) and precision balance (Aczet, CY205C, San Diego, CA, USA), vortex mixer (Thermo Scientific, USA), 2, 15 and 50 mL extraction/conical centrifuge tubes (Thermo Scientific) and Thermo Scientific variable volume Finnpipette (100–1000 µL; 10–100 µL; and 0.5 to 5 mL) were also used.

### 2.2. LC–MS/MS Analysis

An ultra-high-performance liquid chromatography (UHPLC Binary Flex) system coupled with a triple quadrupole mass spectrometer (TSQ Quantis, Thermo Scientific, San Jose, CA, USA) was used for colistin B analysis. To achieve the best sensitivity with symmetrical peak shapes of colistin B, reverse-phase column chemistry, i.e., Hypersil GOLD™ C_18_ (100 × 2.1 mm, 1.9 µm) from Thermo Scientific, San Jose, USA, was used. The column oven and sample manager were maintained at 40 °C and 8 °C, and the injection volume was 10 µL. The mobile phases comprised (A) 1% formic acid in water and (B) 1% formic acid in acetonitrile with a flow rate of 0.4 mL/min. Mobile phase gradient program includes 0–0.5 min, 5% B; 0.5–3 min, 5–50% B; 3–4 min, 50–80% B; 4–5.8 min, 80% B, 5.8–6 min, 80–5% B; 6–9 min, and 5% B phase.

The mass spectrometer was operated with new generation heated electrospray ionization (HESI) in positive mode (ESI^+^). A selective reaction monitoring (SRM) mode was preferred for the analysis of colistin B [13]. The optimized compound-specific selective reaction monitoring transitions with radio frequency (RF) lens voltage and collision energies were determined. The operating ESI parameters (compound independent) included vaporizer temperature (350 °C), ion transfer tube temperature (320 °C), sheath gas (50 Arb), aux gas (10 Arb), sweep gas (1 Arb), spray voltage (3.5 kV). Data acquisition was performed using the SRM function, followed by data processing using TraceFinder 4.1v software (Thermo Scientific, San Jose, CA, USA). For identification and confirmation of the analyte, the SANTE guidelines (2019) were used [14].

### 2.3. Sample Preparation

Test samples were collected from known sources where colistin drug was not applied. The test samples were homogenized by using a mixer and grinder to ensure a small and uniform particle size. A test portion of 3 ± 0.01 g (from homogenized chicken muscle and egg) was weighed in a 50 mL polypropylene centrifuged tube. Then 9 mL of methanol and 9 mL 1% formic acid in water were added to the centrifuged tube; the sample was vigorously shaken and vortexed for about 2 min. This was followed by centrifugation for 10 min at 8500 rpm, and then the 1 mL of supernatant obtained was centrifuged for 10 min at 13,000 rpm and filtered via a 0.20 µm PES syringe filter into a vial (LC) for further analysis. 

### 2.4. Method Validation

The performance of this method was evaluated in terms of specificity, linearity, recovery, precision and limit of quantitation (LOQ) [14,15]. To determine the specificity, blank samples (n = 20) of each matrix were examined and evaluated for intrusions near the colistin B retention times. The linearity was assessed by plotting five levels of calibration curves prepared in pure solvent and matrix. The matrix effect was evaluated by comparing the response of analyte in solvent as well as in a matrix. The accuracy was evaluated by recovery experiments at three different levels and the precision, stated as relative standard deviation (RSD), was ascertained by n = 6 replicates. The LOQ was defined as the lowest concentrations with a signal-to-noise (S/N) ratio of 10:1, and also validated to obtain acceptable recovery (70–120%) with less than 20% RSD. 

## 3. Results and Discussion

### 3.1. LC–MS/MS Analysis

**Figure 1:**
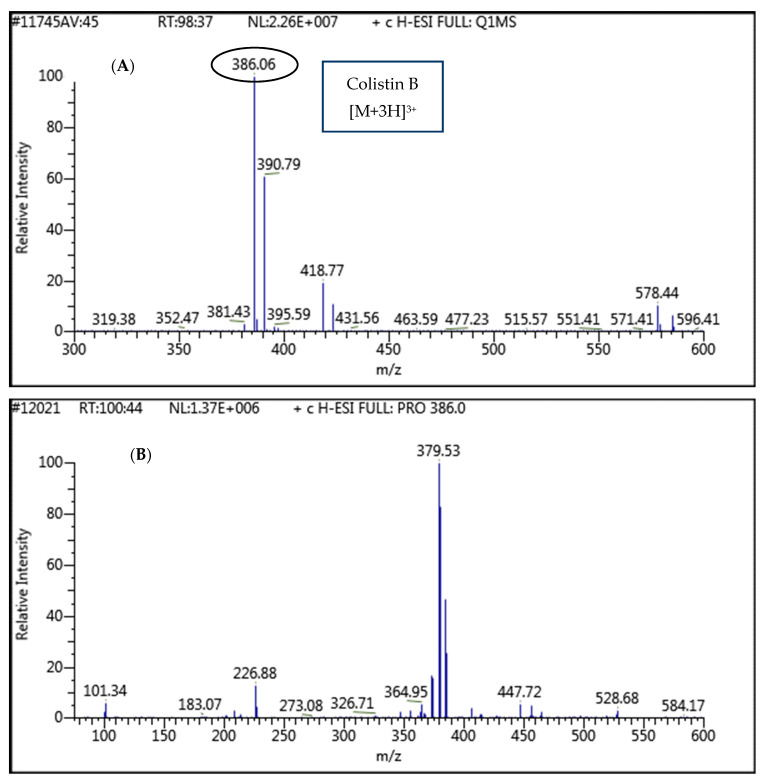

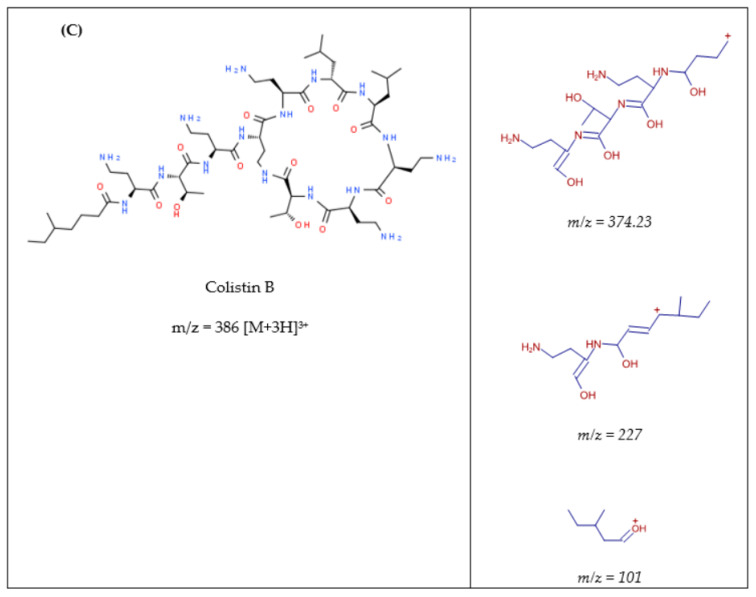
The full MS (**A**) and MS/MS spectra (**B**), and fragmented structures (**C**) for colistin B.

In this work, the mass parameters were optimized with the help of infusing 1 µg mL^−1^ standard solution of colistin prepared in methanol/1% formic acid in water. Data were acquired in a full scan with electrospray ionization (ESI) with positive polarity. The most abundant parent ions were observed with doubly charged ion 578.1 [M + 2H]^2+^ and triply charged ion 386.1 [M + 3H]^3+^. Furthermore, the RF lens voltage was optimized to achieve the best performance of both ions. The most abundant ion *m/z* 386.1 [M + 3H]^3+^ was selected as the precursor ion for further optimization. The parent ion was further fragmented by applying collision energy (argon gas). The full MS and MS/MS spectra for the colistin are represented in Figure 1A–C. The best sensitive and stable fragment/product ions were selected for selective reaction monitoring mode in agreement with earlier reported articles [10,16]. Details of the compound-dependent optimized parameters are listed in Table 2.

Compound-independent (ion source) parameters optimized based on the mobile phase composition showed the best sensitivity at 1 ng mL^−1^ (instrument quantitation limit). 

**Figure 2:**
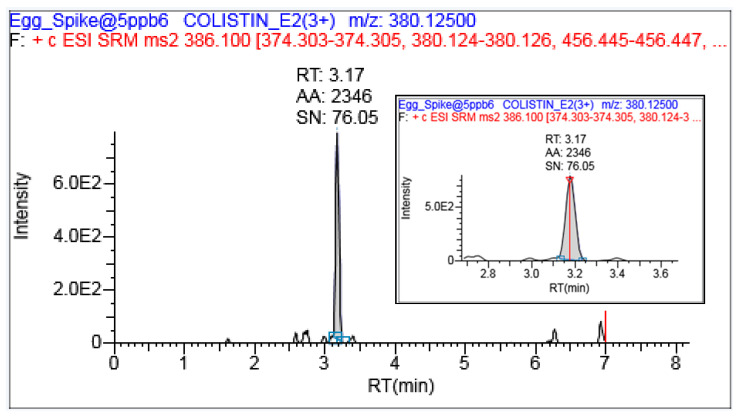
Representative chromatogram for colistin B under the optimized acidified mobile phase. RT: Retantion time; AA: Absolute Area; SN: Single to Nosie Ratio

As colistin is a polar analyte, reverse phase chromatography is preferred in agreement with Fu et al. [16]. The terminal amino group enables more interaction with the C_18_ column chemistry followed by peak tailing. To avoid this, the addition of formic acid was preferred along with acetonitrile solvent, which offered the best ionization efficiency [16]. The gradient program was optimized in such a way that all the polar interference was eluted earlier than the target analyte, and there was no positive signal observed at the observed retention time (3.17). The runtime was extended to 9 min to ensure that the entire matrix interferences coming along with target analytes were completely eluted, and did not have any adverse effect on the next injection (sample/standard). These conditions were able to reduce the matrix effect in both the matrices. In the present study, an acidified mobile phase offered optimum retention, symmetrical peak shape and reproducible signal with retention time (Figure 2) in comparison with Fu et al. [16].

### 3.2. Sample Preparation

By considering the complexity of matrices as well as the physicochemical properties of colistin, a method for sample preparation was developed. It was further modified after studying the core literature using LC–MS/MS for polypeptide antibiotics determination in animal origin food. The published literature for sample preparation primarily discusses the deproteinization step (referred to as acidic pretreatment), performed with the help of organic solvents (acetonitrile and methanol) and acids (trichloroacetic acid and hydrochloric acid) in varied combinations, as shown in Table 3 [2,9,10,11,12,16,17]. 

The developed method is a rapid, simple extraction, which allows users to determine colistin B antibiotic residue in chicken samples at quite low concentration levels. Colistin is a cationic peptide antibiotic with weak acid (carboxylic acid) and base (amino) functional groups. Bladek et al. [2] have reported that colistin could be found in three forms—deprotonated base form, neutral form or protonated acid form. Colistin is highly polar in nature, hence why in the present study polar extraction solvents i.e., methanol and acidified water, were used [18]. In the present approach, the solid-phase extraction (SPE) step was omitted due to tedious processes, long handling time and chances of losing the analyte during sample loading, washing and elution. As methanol and water have good miscibility, there is no phase separation which provides good solubility for colistin. The addition of acidified water is essential to maintain the water level which helps in the matrix dilution. The mixing of methanol and water generated heat due to exothermic reactions and induced better solubility and extractability of the target analyte with less fat interference in comparison with acetonitrile. Acetonitrile carries more fatty content which may interfere with the analysis, followed by disturbance in the column. Without SPE cleanup, the direct injection of methanolic extract was possible due to less fatty matrix co-elution in the proposed protocol. These extraction conditions offered better stability in acidic conditions in both the matrices. The final extract was filtered via a PES membrane filter, which provided a clear and transparent extract by reducing turbidity as compared to other membrane filters (nylon and PTFE). These optimized extraction conditions offered acceptable recovery (70–120%) and precision (<20%). This protocol was also compared with the method of Bladek et al. [2] which used acetonitrile/ammonia-based extraction. There was no significant difference observed in terms of performance (recovery and precision) of the method. The acetonitrile/ammonia-based extraction carried a high matrix background which reduced the S/N ratio for the target analyte by 30–40% as compared to the proposed method. In comparison with previously published reports (Table 3), the proposed method has demonstrated the highest sensitivity of 5 µg Kg^−1^ for eggs and 10 µg Kg^−1^ for chicken muscles. 

### 3.3. Method Validation

#### 3.3.1. Linearity

**Figure 3:**
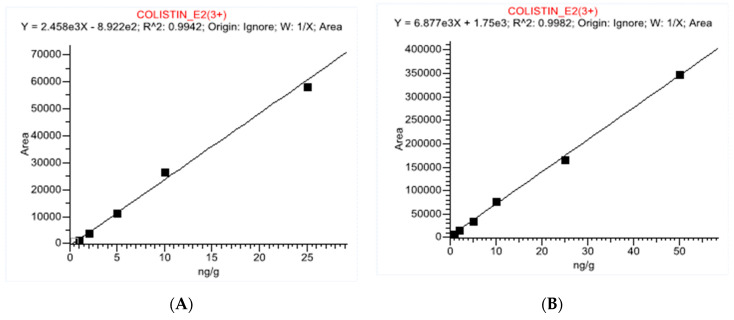
Linearity plot of pure solvent standard used for egg matrix (**A**), and in chicken muscle matrix (**B**).

In this method, the linearity was plotted in the range of 1–25 µg L^−1^ in pure solvent for egg matrix and 1–50 µg Kg^−1^ in chicken muscle matrix (Figure 3). A high linearity was achieved in both the solvent as well as the matrix, with correlation coefficients ≥0.99 and lower than ±20% residuals using a linear equation and 1/x weighting factor, as illustrated in Table 2. 

#### 3.3.2. Limit of Quantitation (LOQ)

**Figure 4:**
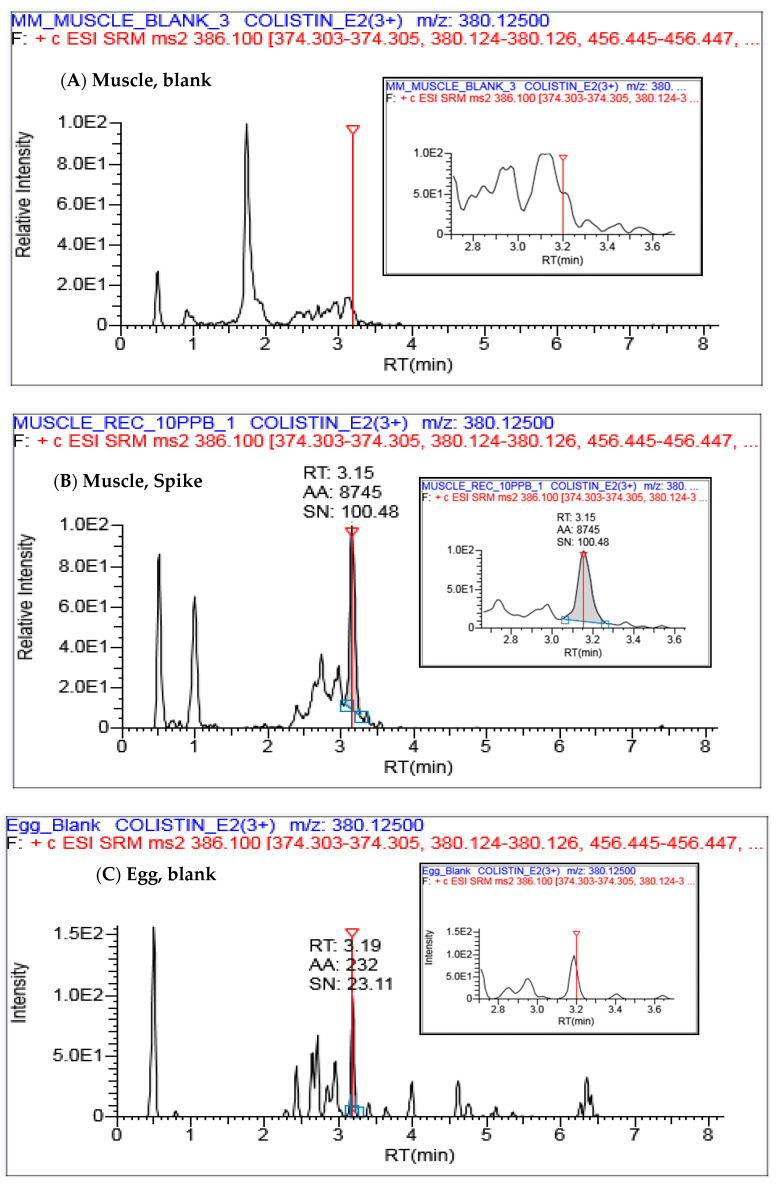

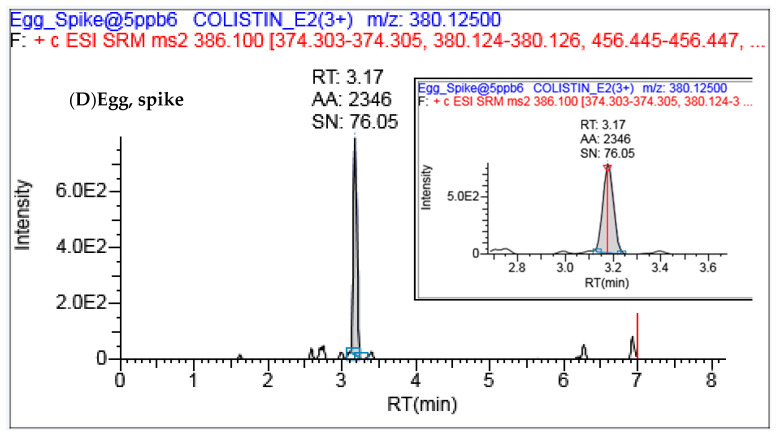
Extracted ion chromatogram (EIC) of colistin B in (**A**) chicken muscle blank, (**B**) 10 µg Kg^−1^ spiked muscle, (**C**) egg blank and (**D**) 5 µg Kg^−1^ spiked egg.

The LOQ value was set at 5 µg Kg^−1^ and 10 µg Kg^−1^ in egg and chicken muscle, respectively (Table 2), which offered acceptable recoveries within 70–120% with <20% RSD for six replicates in both the matrices. The comparison of LOQs observed in the proposed method and earlier reported methods are represented in Table 3. The extracted ion chromatograms (EICs) of colistin B for both matrices at the LOQ level spike and blank matrix are shown in Figure 4. The observed LOQ values were well below the established MRLs, given in Table 1, which help to monitor colistin at a trace level, as it is banned [1]. 

#### 3.3.3. Matrix Effect

The matrix effect observed in eggs was within <±15–20%, and was considered as a low matrix effect within the acceptance criteria of the SANTE guideline, and colistin B quantification was performed using external standard calibration (1–25 µg Kg^−1^) prepared in pure solvent. In the case of the chicken muscle, the matrix effect was observed in the range of 40–55%. To harmonize the results in the muscle, matrix match calibration was preferred in the range of 1–50 µg Kg^−1^. The quantitation of colistin B was successfully demonstrated in both the matrices using this approach. 

#### 3.3.4. Recovery and Precision

The recovery experiment was conducted by use of reference material (RM) to demonstrate the accuracy of this method. In this study, the recovery is assessed through the measurements of additions of known amounts of the analyte at three different levels to a blank matrix, with six replicates for each level. The chromatograms with the lowest spiking level in both the matrices are shown in Figure 4. The average recoveries were observed within 70–94% (3.3–12% RSD) in chicken muscles and 88–107% (2.5–18.6% RSD) in egg samples, which demonstrated the acceptability of the method as per the SANTE 2019 as well as Commission Decision 2002/657/EC guidelines, respectively [14,15]. 

### 3.4. Analysis of Real Samples

Using this method, detection of colistin B was done in real chicken muscles and eggs. Sampling was carried out for 50 samples (30 eggs and 20 muscles) based on a non-probability approach in Solan, Himachal Pradesh, India. All the samples were extracted as per the stated protocol and analyzed using LC–MS/MS under the above optimized conditions. Out of 20 muscles, three samples had a colistin B concentration range of 50–560 µg Kg^−1^. However, the egg samples did not show the presence of colistin B in them. 

## 4. Conclusions

This article represents a simple, robust, cost-effective and sensitive sample preparation protocol followed by LC–MS/MS analysis of colistin B in chicken muscles and eggs. The proposed method has been validated and established for its specificity, accuracy and suitability for routine and repetitive analysis in chicken products for regulating colistin utilization. The unregulated colistin usage in animal husbandry has prompted the evaluation of the presence of colistin sulfate residue in both tissues and chicken feed, so that an effective measure can be developed. Additionally, the monitoring of colistin sulfate in both tissues and chicken feed will enforce the regulated use of colistin (feed additives) during food animal production. Based on the present study, a highly economical and simple method for colistin B extraction is developed in difficult matrices like chicken muscles and eggs, without the use of SPE clean-up procedures. This proposed protocol provides a cost-effective solution for food testing labs by reducing 60% cost of the sample preparation along with the time required for SPE cleanup. The developed protocol can also increase the throughput of commercial food testing labs.

## Figures and Tables

**Table 1:** Maximum residue limits (MRLs) of colistin recommended by different regulatory agencies, adapted from Kumar et al. [1].

Target Tissue	MRLs (µg Kg^−1^)	Regulatory Agency
Muscle, Egg	150, 300	EU
Muscle, Egg	150, 300	FSSAI
Muscle	150	MAC
Muscle	150	PHR Hong Kong

EU—European Union; FSSAI—Food Safety and Standards Authority of India; MAC—Ministry of Agriculture China; PHR Hong Kong—Public Health (Animals and Birds) (Chemical Residues) Regulation Hong Kong.

**Table 2:** Ultra-high-performance liquid chromatography–tandem mass spectrometry (UHPLC–MS/MS) parameters for colistin B and its validation results in chicken muscle and egg.

Adduct	Precursor ion (*m/z*)	Product ions (*m/z*)	RF Lens (V)	CE (eV)	Calibration Range	Linearity	LOQ (µg Kg^−1^)	% Recovery (RSD %)		
Muscle								10 µg Kg^−1^	50 µg Kg^−1^	100 µg Kg^−1^
[M + 3H]^3+^	386.1	374.3	122	12.12	1–50 ng mL^−1^	0.998	10	71.1 (12.1)	70.7 (6.6)	93.8 (3.3)
[M + 3H]^3+^	386.1	380.1		10.52						
[M + 3H]^3+^	386.1	227.1		15.83						
[M + 3H]^3+^	386.1	101.1		15.98						
Egg								5 µg Kg^−1^	10 µg Kg^−1^	150 µg Kg^−1^
[M + 3H]^3+^	386.1	374.3	122	12.12	1–25 ng mL^−1^	0.994	5	88.6 (18.6)	107.5 (11.5)	105.5 (2.5)
[M + 3H]^3+^	386.1	380.1		10.52						
[M + 3H]^3+^	386.1	227.1		15.83						
[M + 3H]^3+^	386.1	101.1		15.98						

CE—collision energy; RF—radio frequency; LOQ—limit of quantitation; RSD—relative standard deviation.

**Table 3:** Comparison of earlier reported sample preparation protocols used for colistin B detection with newly proposed protocol.

Sample Type	Sample Weight (g)	Extraction	Clean Up	Evaporation Used	LOQ (µg Kg^−1^)	References
Muscle, Egg	2	Acetonitrile, Water and Ammonia	NA	Nitrogen	10	[2]
Bovine Liver, Kidney	0.5	Trichloroacetic Acid, Acetonitrile, and Formic Acid	OASIS^®^ HLB Sep-pak	Nitrogen	50	[9]
Bovine Muscle, Liver	2.5	Hydrochloric Acid	Phenomenex Strata-X cartridges	Nitrogen	2/5 (MRL)	[10]
Fishes	5	Methanol, Water, and Hydrochloric Acid	ProElut PLS	Nitrogen	40	[11]
Chicken Muscle	5	Trichloroacetic Acid, Methanol, Water and Hydrochloric Acid	IRIS Polymeric	Nitrogen	50	[12]
Porcine Muscle, Liver, Kidney; Bovine Muscle, Liver, Kidney; Chicken Muscle, Liver, Kidney, Egg; Sheep Muscle	2	Trichloroacetic Acid, and Acetonitrile	Oasis WCX	Nitrogen	5–30	[16]
Bovine Muscle	3	Ammonium Acetate, EDTA, Trichloroacetic Acid and Sodium Chloride	Sep-PAK WCX	NA	33	[17]
Chicken Muscle, Egg	3	Methanol, Water and Formic Acid	NA	NA	10 and 5	Present Study

NA—not applicable.
